# Design and rationale for evaluating the impact of salad bars on elementary school students’ fruit, vegetable, and energy intake: a wait list control, cluster randomized controlled trial

**DOI:** 10.1186/s12889-022-14744-y

**Published:** 2022-12-09

**Authors:** Melanie K. Bean, Hollie A. Raynor, Laura M. Thornton, Lilian de Jonge, Suzanne E. Mazzeo

**Affiliations:** 1 Department of Pediatrics, School of Medicine, Children’s Hospital of Richmond at Virginia Commonwealth University, Box 980140, Richmond, VA 23298 USA; 2 Department of Nutrition, University of Tennessee, Knoxville, 1215 W. Cumberland Ave, Knoxville, TN 37996 USA; 3 Department of Psychiatry, University of North Carolina, Chapel Hill, NC 27599 USA; 4 Department of Nutrition and Food Studies, 10349 Democracy Ln, Suite 306, Fairfax, VA 22030 USA; 5 Department of Psychology, Virginia Commonwealth University, Box 842018, Richmond, VA 23284 USA

**Keywords:** Salad bar, Elementary school, Dietary intake, National School Lunch Program, Fruit and vegetable intake

## Abstract

**Background:**

Most children do not consume the recommended amount of fruit and vegetable (FV) servings. Changing the school food environment can be a cost-efficient, effective approach to improving children’s dietary quality. There is great popular support for school salad bars as a means to increase children’s FV intake within the National School Lunch Program (NSLP), yet empirical research is limited. Further, although FV consumption can facilitate healthy weight management if these foods replace high calorie items, there is a need to enhance understanding of salad bars’ influence on children’s diet quality and energy intake within the NSLP. This is particularly important to investigate in schools in communities characterized by high poverty, as students they serve are particularly likely to rely on school meals.

**Methods:**

This report describes the design and rationale of a federally-funded investigation that uses validated methods to evaluate school salad bars. This district plans to install salad bars into 141 elementary schools over 5-years, facilitating the conduct of a waitlist control, cluster randomized controlled trial. Specifically, 12 pairs of matched schools will be randomly selected: half receiving a salad bar (Intervention) and half serving pre-portioned FVs only, standard under the NSLP (Control). Thus, groups will have different FV presentation methods; however, all schools will operate under a policy requiring students to take at least one FV serving. Schools will be matched on Title I status and percent of racial/ethnic minoritized students. Intake will be objectively assessed at lunch in each school pair, prior to (baseline), and 4–6 weeks after salad bars are installed (post), yielding ~ 14,160 lunch observations throughout the study duration. Cafeteria sales and NSLP participation data will be obtained to determine how salad bars impact revenues. Finally, implementation factors and cafeteria personnel’s perspectives will be assessed, to identify barriers and facilitators to salad bars use and inform sustainability efforts. Proposed methods and current status of this investigation due to COVID-19 are described.

**Discussion:**

Results will have great potential to inform school nutrition policies and programs designed to improve dietary quality and reduce obesity.

**Trial registration:**

Retrospectively registered (10/28/22) in clinicaltrials.gov (NCT05605483).

## Background

Fruits and vegetables (FVs) are essential components of a healthy diet and can reduce risk for many chronic illnesses; [1, 2] yet, most children do not consume the recommended number of servings of these foods [3–5]. This is concerning, as inadequate FV intake is linked to increased risk for cardiovascular disease, [1, 2] and certain cancers [6]. Children from minoritized backgrounds living in communities characterized by high poverty have the lowest FVs intake [7]. These children often live in food deserts with limited FV affordability and access [8, 9]. They are also the most likely to participate in the National School Lunch Program (NSLP), [10] making the school food environment a critical target of public health efforts to enhance dietary quality [11, 12].

A major goal of the NSLP is to increase FV intake, with a long-term aim of reducing obesity, [12] yet it is unclear how increasing children’s FV intake relates to energy intake in the NSLP [13]. Greater FV consumption could decrease energy intake if these foods replace higher-calorie items; however it could increase energy intake if FVs are merely added to regular meals [13–16]. Understanding how FV consumption influences energy intake in school lunch is particularly important to examine in schools serving predominantly minoritized children living in communities characterized by high poverty, as this population faces disproportionately high obesity risk [17] and is most likely to be impacted by school food policies, given its reliance on the NSLP [10].

Salad bars are promoted as a strategy to increase students’ FV intake, variety, and choice within the NSLP [18–22]. For example, the Chef Ann Foundation raised >$15.8 million and donated 6,083 salad bars to schools, with 260 schools on a waiting list to receive one [23]. The Centers for Disease Control (CDC) notes that it supports the mission of *Salad Bars to Schools* (a public-private partnership which includes the Chef Ann Foundation, the Whole Kids Foundation, and government programs) as a strategy, “to ensure every child has the choice of healthy fruits and vegetables each day at school.” [23]. Yet, rigorous evaluation of school salad bars to determine their impact on dietary intake patterns is needed to ensure they meet their intended purpose.

Increasing accessibility to a variety of FVs and fostering choice are two mechanisms proposed to explain how salad bars might positively impact dietary consumption [19–22, 24–27]. However, the limited research investigating this relation has yielded somewhat mixed results. For example, results of one study indicated that greater variety of FVs offered (independent of salad bar status) was associated with higher FV intake among elementary school students [22]. Similarly, the introduction of salad bars into Title I schools was associated with increases in the variety of FVs offered and selected [28]. Yet, self-served *portions were smaller* and FV intake *decreased* [28].

Fostering choice might be an especially helpful in promoting FV intake in children. Indeed, in one study conducted with 4th and 5th grade students (*N* = 1193), 85% reported that they liked having the option to choose FVs from their school salad bar; however only 44% said they used the salad bar at least once per week [28]. A limitation of that investigation was that salad bars were offered *in addition* to fixed portions of FVs on the lunch line, increasing variety, yet precluding isolation of presentation methods; this design also introduced confounds related to FV familiarity (e.g., of canned [29] and/or heated FVs) competing with salad bars.

In addition to fostering choice, scholars have posited that salad bars might impact dietary consumption by increasing FV intake, and decreasing overall caloric consumption, via energy displacement [30]. Yet it is unclear how increasing FV intake affects children’s overall energy consumption, in part due to methodological limitations of extant research. [16, 31, 32]. Even less is known about how increasing FV intake within the NSLP (which limits calories available per meal) influences energy intake at lunch. One of the few existing studies in this area found that increased lunch FV intake was associated with decreased self-reported energy intake in students at schools with salad bars [33]. In contrast, a subsequent study [16] found that students who consumed the most FVs at lunch had the highest total energy intake. However, non-FV energy decreased across FV intake groups (thus at least some energy consumed from other sources was displaced by FVs). This study was conducted prior to the current NSLP guidelines, and only 54% of the schools it evaluated had salad bars. In a cross-sectional investigation conducted under the current NSLP, students in schools with salad bars consumed more energy from vegetables compared with students from schools without salad bars. Yet, evidence was inconsistent regarding FV displacement of other lunch calories [34].

Other limitations of previous research include a lack of longitudinal and objective data [35]. For example, in multiple cross-sectional studies, middle and high school students self-reported greater FV intake in schools with salad bars [36–38]. However, because dietary intake was assessed via self-report only it is more subject to bias than objectively measured consumption. In contrast, 1st -5th grade students’ FV intake (assessed via objective plate waste) was no higher in schools with salad bars compared with schools serving pre-portioned FV only [22]. However, this latter work was conducted > 15 years ago and might not be generalizable to today’s children, particularly given significant changes to the NSLP. There are also several program evaluations available online, although methodological concerns limit their internal validity (e.g., lack of comparison groups, post-only assessments, or low validity of FV assessment methods) [35].

Only two quasi-experimental studies [28, 33] have prospectively examined the impact of salad bars on dietary intake among elementary school students, and they yielded conflicting results. Moreover, only one of these investigations, [28] was implemented under the current NSLP standards and assessed dietary intake objectively. In the first of these studies, Slusser et al. [33] compared FV intake before and after salad bar installation in three schools. FV intake increased by 1.12 servings per day, as measured by 24-hour recalls; however, objective assessments of FV consumption were not conducted. There was also a 2-year gap between baseline and post assessments (and a 30% student transience rate), introducing potential history effects and reducing the likelihood that the same children were assessed at both time points. Bean and colleagues recently assessed FV intake before and one month after salad bars were installed in two Title I elementary schools serving predominately Black children, all of whom received free meals [39]. Using objective, digital imagery plate waste methods, they found that students selected significantly more *types* of FVs after the introduction of salad bars. However, at post, self-served FV portions were significantly smaller than those served by food service personnel, and mean FV intake *decreased* by 0.65 cups (c), compared to when FVs were pre-portioned exclusively [28]. These results suggest that increasing access to FVs might not be sufficient on its own to shape consumption patterns in this population. Importantly, neither of these prior studies included comparison groups.

Bean and colleagues subsequently compared FV intake in schools with salad bars with matched schools serving proportioned FVs only, within this same district. Although there was some evidence that vegetable consumption was higher in salad bar schools, different patterns of FV selection and consumption were observed across school pairs, suggesting that school environment factors other than salad bar access influenced FV intake [40]. Importantly, salad bars in this district were offered *in addition to* pre-portioned FVs on the serving line, and salad bar usage varied widely (8–64%) between schools. The continued availability of pre-portioned FVs precluded the ability to isolate the effects of salad bars on dietary consumption. There were also between-school differences in salad bar location, a factor demonstrated to impact usage [41]. These results highlight the need for rigorously-designed, prospective evaluations of school salad bars, that include larger numbers of schools and comparison groups, use robust scientific methods, assess dietary intake objectively, and place salad bars in a consistent location.

Finally, salad bars are also often proposed as a mechanism to enhance NSLP participation [35]. NSLP participation is a priority across districts, as it enhances the economic stability of school nutrition departments [42]. Greater NSLP participation could also have positive public health implications, as school meals offer superior nutrition, compared with meals brought from home [42]. Despite these benefits of NSLP participation, it has consistently declined over time [43]. Moreover, there are no empirical data to support the claim that salad bars increase participation in the NSLP. The current study will examine how salad bars impact cafeteria sales and NSLP participation within a diverse district, with wide variations in NSLP participation, enhancing generalizability of findings related to these critical revenue sources.

In sum, there is an urgent need to improve the quality of children’s dietary intake. Optimizing school meals within the NSLP can have a significant public health impact. Tremendous resources have been invested in school salad bars as a means to increase FV intake, yet it is not known if they achieve this goal. By randomly selecting schools receiving salad bars, matching them with those serving pre-portioned FV only, and conducting a comprehensive, longitudinal evaluation that includes objective, validated assessments of dietary intake, this investigation will address these critical gaps. Specifically, this study will identify: (1) how salad bars impact dietary consumption in NSLP lunches, and (2) consequences of, and barriers and facilitators associated with, salad bar implementation. It will also examine the potential moderating role of the school-level sociodemographic factors of percent high obesity risk racial/ethnic minority students and Title I status on dietary consumption, and salad bar implementation barriers and facilitators. In the current study, all FVs on the salad bars will be fresh and *replace* all fixed portion FVs on the serving line. All schools will operate under a policy requiring students to take at least one FV serving [44]. This will allow examination of the independent and combined impact of variety and choice, two mechanisms with potential to increase FV intake, within the NSLP. We hypothesize that schools with salad bars will manifest greater increases in both FV selection and consumption, and decreases in FV waste, compared with schools without salad bars. In addition, we hypothesize that schools with salad bars will have greater improvements in dietary quality and reductions in total energy intake at lunch, compared with schools without salad bars.

Data from this trial will yield some of the clearest evidence related to salad bars to date, providing a strong evidence base to evaluate school nutrition policies designed to enhance dietary intake and reduce health disparities within the NSLP. Results can guide resource allocation and inform targeted interventions and policies designed to reduce obesity.

## Methods and design

### Study setting

This study will be conducted in a large public school district in the Mid-Atlantic United States. This district plans to install salad bars in all 141 elementary schools (K-6th grades). Prior to the onset of the current trial, 50 salad bars had already been installed. There are thus 91 schools remaining for potential inclusion in the proposed investigation, with salad bars to be installed over a 3-year period. Salad bars are launched throughout the year. This installation schedule provides a unique opportunity to evaluate a natural experiment, with a waitlist control design. Specifically, randomly selected schools receiving salad bars will serve as Intervention schools and be matched with Control schools serving pre-portioned FVs only. These waitlist (Control) schools will receive salad bars in the subsequent school year. Thus, this investigation offers a unique and *time-sensitive* opportunity to conduct a waitlist control, cluster randomized controlled trial.

### Design

Twelve pairs of matched schools will be randomly selected: half will receive a salad bar (Intervention; replacing all other FVs) and half will only serve pre-portioned FVs, standard under the NSLP (Control). Thus, groups will have different FV presentation methods; however all schools will be operating under a policy requiring students to take at least one FV serving [44]. In contrast to prior salad bar research, [28] salad bars in the current study will replace all other FVs on the lunch line, and students can select their entire meal from the salad bar. This implementation model facilitates a more rigorous comparison between schools with and without salad bars than that conducted in prior research. Schools will be matched on Title I status (a proxy for socioeconomic status [SES]) and percent of racial/ethnic minority students based on higher obesity risk. Dietary intake at lunch will be objectively assessed in each pair of schools, prior to (baseline), and 4–6 weeks after salad bars are installed (post), resulting in ~ 14,160 lunch observations throughout the study duration. The primary dependent variable of interest is change in FV intake, assessed using digital imagery plate waste methods. Cafeteria sales and NSLP participation data will also be examined to assess the potential impact of salad bars on these critical revenue sources. Finally, we will assess implementation practices and cafeteria personnel’s perspectives, to identify barriers and facilitators to salad bars and inform sustainability efforts.

Prior to random selection, each school will be assigned to one of 4 categories based on sociodemographic variables. We used information from schools that received salad bars in the first 2 years of the district’s program (prior to the current study) to develop matching procedures. Matching was based on: 1) Title I status, which includes % free and reduced-price lunch, and 2) % of students from racial/ethnic backgrounds at higher risk of obesity (Native American, African American/Black, Latinx, Native Hawaiian/Pacific Islander, more than one race). Four categories of schools resulted: A = < 40% minoritized AND not Title I; B = 40–60% minoritized AND not Title I; C = 40–60% minoritized AND Title I; and D = > 60% minoritized AND Title I. Matching procedures were then applied to the remaining 91 schools, demonstrating that each category has adequate numbers of schools in each year of this study to successfully execute the proposed aims.

**Fig. 1:**
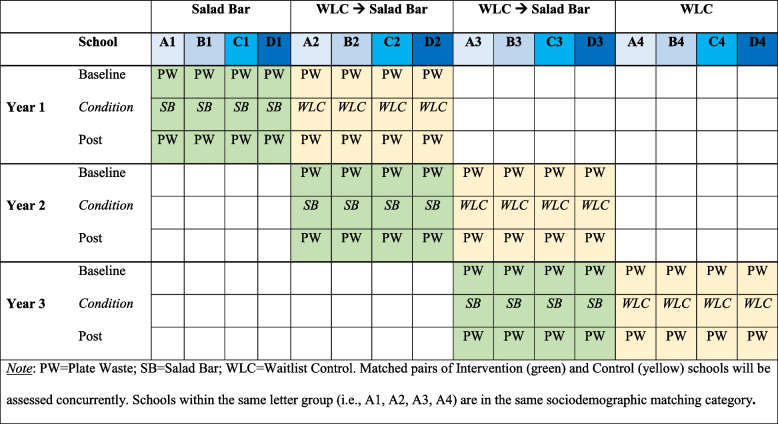
Design of wait-list control randomized controlled trial of school salad bars

As illustrated in Fig. 1 and 16 different schools will be included (A1➔D4), 4 from each sociodemographic category. In Year 1, 8 schools will be randomly selected: 4 with salad bars (A1➔D1; Intervention), matched with 4 serving pre-portioned FVs only (A2➔D2; Control). In Year 2, these 4 control schools will receive salad bars and become Intervention schools, and will be matched with 4 new, randomly selected Control schools (A3➔D3). These procedures will be repeated in Year 3, with A3➔D3 becoming Intervention schools, matched with new randomly selected Control schools (A4➔D4). There will ultimately be 12 pairs of matched schools (12 Intervention [shaded green in Fig. 1] and 12 Control [shaded yellow]).

Each pair of Intervention and Control schools will be assessed concurrently, minimizing the threats maturation and history effects pose to internal validity [45]. Baseline and post-assessments within each pair will be conducted on days with the same menu cycle whenever possible, which controls for potential confounds, such as the varying palatability of meals and seasonal variations in food availability (the district’s schools have 5 week menu cycles). We will also control for month of assessment in all analyses. This is particularly important in the elementary school setting, where students are continually adjusting to the environment and school breaks can affect eating habits [46]. Inclusion of baseline assessments will allow us to control for any baseline differences between schools. Raters will be masked to avoid potential bias. Moreover, conducting independent double ratings of ~ 20% of meals will further minimize the influence of any potential bias. Finally, this study’s internal validity is enhanced by matching schools on key variables prior to randomization, so that they differ primarily on presence of a salad bar [45]. Matching procedures were carefully developed to evaluate how school-level factors of race/ethnicity and SES might moderate the impact of salad bars. Other variables that could potentially vary between schools and impact outcomes will be carefully monitored and considered in analyses (e.g., NSLP participation or school environment factors). Plate waste assessments will be conducted in wait list control schools during two consecutive years; however, students will not know the purpose of the assessments, and their prior experience with these (minimally obtrusive) methods is not likely to alter dietary behaviors during the following school year.

### Target population and sample

The school district included in this study serves > 189,000 elementary school students (K-6th grades); 48 of its 141 elementary schools are Title I, and 31% (> 58,500) of students receive free and reduced-price lunch (1-91% across schools). Students are 40% White, 25% Latinx, 19% Asian, 10% African American/Black, and 5% more than one race. These data highlight the great diversity of this district with respect to SES and race/ethnicity, making it an ideal setting in which to examine if the potential differential impact of salad bars based on these school-level factors. Potential schools have a mean enrollment of 692 students and mean NSLP participation rate of 53% (range 23–85% [national rate is 61%]) [43].

Lunches from all children who participate in the NSLP on rating days will be eligible. We conservatively estimate rating ~ 295 lunches (~ 80% of NSLP participants) per school on each assessment date, from K-6th grade students, with equal grade distributions. This rate is consistent with those achieved in our formative work [28]. We thus anticipate rating 7,080 lunches (3,540 per condition) at baseline and post-assessments across 12 pairs of matched schools, for a total of ~ 14,160 school lunch observations throughout the study duration. To inform understanding of barriers and facilitators to salad bars, cafeteria personnel from target schools will complete weekly logs and post-surveys assessing their perspectives related to salad bars; there are 4–7 staff per school, from diverse backgrounds. With an 80% response rate, consistent with rates we achieved previously, [47] we anticipate that ~ 70 cafeteria staff will participate.

The district’s Food and Nutrition Services staff conduct a standardized training for all students and staff prior to their salad bar launch. Student training includes informing students that salad bars will replace all other FVs on the lunch line, describing what foods will be offered, and instruction related to proper salad bar use (e.g., sanitation, food safety and handling, and portion and food group guidelines). Trainers also provide education related to the importance of consuming FVs, developmentally tailored based on grade-level. Staff training includes strategies to ensure USDA adherence related to portion sizes and food groups, monitoring and implementation protocols, menus, food preparation, storage and handling, and cashier training. Cashiers are trained to ensure meals adhere to NSLP guidelines (e.g., ≥½c FV), with strategies taught to facilitate visual estimation of this portion.

All students encounter the salad bars as the first option in the lunch line. This placement is consistent across schools and intentional, as placing healthier foods first in line increases their selection [48]. Further, Adams et al. reported that FV selection and consumption was more than 4 times greater in schools with salad bars located within the serving line, prior to the point of purchase, compared with those located outside the line, after the point of purchase [41]. This district’s salad bars include FVs (7 options [all fresh] that rotate based on seasonal availability) and 2 proteins, with the option to also select a whole grain and milk from the second part of the line. Salad greens and dressing (1oz serving) are always offered. Salad bar menus are consistent across schools. FV portions must adhere to USDA serving size guidelines for the NSLP. To facilitate meeting these requirements, lunch trays have an identified “square” to fill with any combination of FVs desired. Signage also facilitates adherence with NSLP guidelines (e.g., use of colored stars for each food group). In addition, cafeteria staff monitor salad bars on the line to ensure students serve adequate FV portions. If students’ trays do not meet USDA requirements, staff guide them back to the salad bar to select additional fruit or vegetable options before ringing in the meal. Once installed, salad bars replace all other FVs on the serving line, thus students must select FVs from the salad bar. Moreover, students can choose to select their entire meal from the salad bar and bypass the hot meal completely. Thus, in the current investigation, we will be able to investigate how FV presentation (self-serve vs. fixed portions) and location impact dietary intake, within the NSLP.

### Cafeteria assessor and digital imagery (DI) rater training

Masked cafeteria assessors and DI raters will be trained following detailed protocols used in previous research, which yielded excellent inter-rater reliabilities (IRRs), maintained throughout the trial [28, 40].

Cafeteria assessors will participate in a standardized training that includes details of cafeteria procedures, in addition to extensive practice taking photographs from a standard angle (45°) and distance using mock trays. Feedback will be provided until methods are consistently applied.

Independent raters (masked to timepoint and study hypotheses) will be trained according to protocols to rate item selection and consumption from the photographs. Raters will view multiple images of plated and post-consumption trays from previous salad bar investigations. They will indicate the % of each item consumed in 20% increments [49–51]. Because of the variable reference portions from salad bars, raters will be carefully trained to assess portion sizes and volume visually (to the nearest ¼c) for different servings of FVs (that had been previously measured) using photographs and standard portions as a guide, consistent with our previously validated methods [52]. Raters must achieve IRRs (assessed via ICCs) of ≥ 0.80 for all items evaluated, as well as ICCs ≥ 0.80 when compared with “gold standard” ratings to indicate readiness to participate in the study.

## Measures

### Cafeteria environment

Prior to baseline, we will obtain details about each school via systematic observations and reports from the district’s Food and Nutrition Services to include: (1) number of points of sale, (2) lunch period length (if different from 30 min district standard), (3) lunch time per grade, (4) presence of a school garden, (5) other relevant programs (e.g., Farm to School programming); (6) free and reduced-price lunch and NSLP participation rates; (7) other food environment aspects that might vary between schools. These factors will be considered in analyses as potential explanations of any differential impact of salad bars across schools.

### Demographics

School, sex, and grade will be obtained from labels affixed to trays (See Procedures). On rating days at each school, study staff will obtain updated enrollment data, number of students absent per grade, number of lunches brought from home per grade based on the average of two rater counts, and number of student and parent opt-outs (to determine study participation rate).

### FV selection

Raters will note which items were available and selected, including number and types of FVs (coded with a location). Vegetables will be categorized as dark green, red/orange, legumes, starchy, and other, consistent with USDA/NSLP [44]. Fruit is defined as whole fruit (not juice). *Variety* will be scored as the number of different types of FVs and as the number of different categories of vegetables the student selected. *Location* (salad bar, lunch line, or point of sale) and serving type (self-serve, pre-portioned) of each FV will be obtained from photographs of the lunch line.

### Plate waste

Raters will estimate the % of each item consumed in 20% increments. Visual stimuli (pie charts) on a validated tick sheet assist raters in making judgments [53]. Ounces remaining (to the nearest 0.5oz, assessed in the photographs of measuring cups) will be used to determine % consumed for beverages, consistent with methods applied previously [49]. Plate waste (%) and % consumed will be determined for (1) FVs, (2) non-FVs, and (3) beverages. The average % consumed for all salad bar components will be calculated and applied to dressing (if used) to calculate consumption. These methods are consistent with those used in our prior salad bar investigation, yielding excellent IRRs [40].

### Nutritional information

Recipe data from standard portions (average of 3 measured portions) and all items from the salad bar will be entered into the Nutrition Data System for Research (NDSR) [54, 55] for analysis. The amount missing will be assumed to have been consumed, [16, 49, 56] and subtracted from the plated portion. FV, non-FV, beverage, and total energy (kcal) available and consumed will be calculated. The Healthy Eating Index (HEI) 2015 [57] will assess dietary quality of the lunch; scores for each component will be calculated from NDSR data, [57] and a total HEI score (possible range = 0-100) for the meal will be created. The HEI 2015 was developed by the USDA and the National Cancer Institute to evaluate dietary quality compared with current USDA guidelines.

### Sales data

Monthly NSLP participation rates, FV sales/reimbursements, and salad bar sales/reimbursements (including whether the salad bar was part of a NSLP meal or a la carte [Intervention schools]), and number of days lunch was served per month will be obtained from the district for all selected schools.

### Cafeteria staff survey

At post-testing, cafeteria staff will be asked to complete a survey based on our formative work [47] and in previous investigations [58–60] to assess perceptions of the school food environment, perceived barriers and facilitators to salad bars, satisfaction with staff and student training, impact of salad bars on daily work, and suggestions for improvement. Responses will identify barriers and facilitators to salad bar programs to inform their sustainability and will be considered as potential factors explaining any differential findings between schools. Surveys will be anonymous and available in the preferred languages of each cafeteria personnel. To increase participation, respondents will have the option to enter a raffle for a $25 gift card. These methods yielded high response rates in our prior work [47].

### Process logs

Cafeteria managers at the target schools will complete process logs for 4 weeks after salad bars are installed, assessing implementation practices (e.g., location of salad bar, monitoring, menu) and any aberrations from protocols. Managers will receive a $25 gift card for completion of logs. Food and Nutrition Services staff will provide any modifications to salad bar training or methods. We will also monitor any changes to NSLP or school/district policies that might impact the current investigation for consideration in analyses.

## Procedures

### Consent

Passive consent will be used; students can refuse to have their tray imaged without penalty. Parents/caregivers will be informed of the study via notification letters sent home by the schools, with a form to return if they wish to opt their child out of assessments. On rating days, teachers will read a short script to students prior to lunch to inform them about ratings occurring in the cafeteria, noting that participation is voluntary.

### Cafeteria procedures

**Fig. 2:**
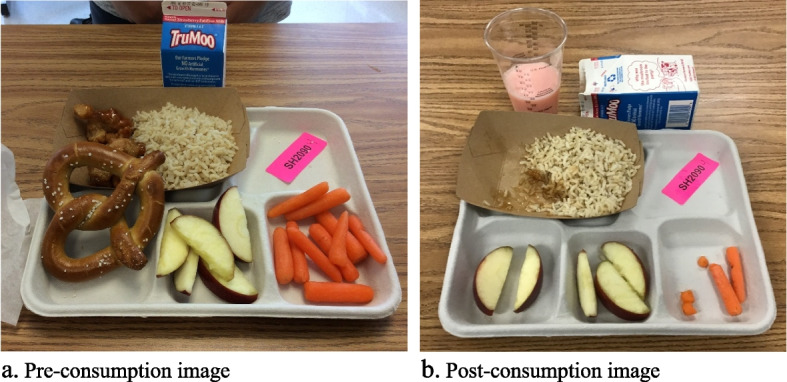
Pre-consumption (**a**) and post-consumption (**b**) image of a school meal using digital imagery methods

Lunchroom observations will be conducted in randomly selected, matched pairs of schools by trained assessors (typically graduate and undergraduate students in psychology, nutrition, or public health). Each matched school pair will be rated concurrently, before (baseline) and 4–6 weeks after salad bars are installed (post), for menu consistency. Lunch selection and consumption will be assessed using the validated DI methods described, [49, 51, 61] and implemented successfully in our prior work [28, 52] . As students enter the lunch line (in grade groups), staff will obtain their assent. If students agree, they will affix a label on their tray (with grade recorded). Labels are color-coded and numbered to track sex and facilitate subsequent rating by matching pre- and post-consumption images. As students exit the lunch line, research staff will place their tray on a reference table to standardize the distance and take a photograph. All photographs will be taken digitally with iPads at a standard distance and ~ 45° angle from the meal [16, 62, 63].  The number of students who bring a lunch from home per grade will be independently counted by two raters and an averaged used. If food from home (typically drinks and snacks) is added to the school meal, it will be included in the photograph and subsequently rated if it is visible at both pre- and post-consumption. Trash cans will be moved during observations, except for those staffed by raters. Upon completion of each lunch period, students will be instructed to leave their trays on the lunch table. Staff then prepare each tray for rating. They reposition items to ensure the label and all items are visible. Beverage waste is poured into a clear measuring cup to facilitate rating  [63]. Containers for unopened beverages are left unopened and positioned upright; containers for empty beverages are placed on their side. If opened, pre-packaged items (e.g., chips) are removed from packages. We developed this method in our formative work to facilitate estimation of intake in the lab and overcome lower reliabilities found for packaged food in a prior plate waste study [64]. Staff will then take another image documenting what was left unconsumed. Trays are then discarded and tables cleaned for the next lunch period. See Fig. 2. On rating days, staff will make detailed process observations and two raters will independently count the number of lunches brought from home (by grade).

Images will be subsequently uploaded onto computers in the lab to prepare for rating. After post-ratings, staff will distribute FV-themed pencils or other small incentive to all children (regardless of participation), as tokens of appreciation. We have found that ~ 10–15 staff per school (to administer labels, take photographs, prepare trays for imaging, and clean-up) optimizes rating efficiency and minimizes disruptions to cafeteria flow (an extremely high priority for school staff). Benefits of DI are the rapid acquisition of data in the cafeteria environment (< 5 s per tray), [63, 64] low staff and school burden, and ability to conduct objective ratings in an unhurried laboratory setting. Moreover, both rater agreement and validity for estimating portion sizes and waste using DI methods, including those from salad bars, are very high [52, 62, 63].

On each rating day, study dietitians will verify all foods against school menus. Photographs of the lunch line will be taken to document placement of FVs, including salad bars. Photographs and direct measurements (using a calibrated food scale) of 3 portions of each item will be taken and an average used as the reference for a standard portion. Serving sizes, product information and recipes of all items offered will be obtained from the district’s dietitian after each rating period, for entry into NDSR and subsequent analyses.

### Salad bar reference portions

Given variable portions of self-serve salad bar items, we will use methods described previously [52] to create reference portions of all salad bar menu items. Food will be prepared in the lab, using methods consistent with those used in the schools (e.g., diced, sliced, or whole). Two dietitians will independently serve 3 portions of each item (½c, ¼c, ¾c, and 1c) onto cafeteria trays. Portions will be weighed in triplicate (after taring to remove the weight of the tray), and an average used as the reference weight. Staff will take photographs of these portions for use by DI raters when estimating starting portions from salad bars.

### DI rating procedures

In the lab, trained, masked, independent raters will simultaneously view the images of the food selection and waste for each tray on a computer screen [49–51]. Raters will record which items were selected and then estimate the % of each item (FVs, non-FVs, beverages) left on the plate. Given variable reference portions for salad bar items, raters will estimate the plated volume of FVs selected to the nearest ¼c, consistent with prior research [13, 65]. Reference images of each salad bar item in standard portions will be available to assist raters in making judgements. Salad dressing usage (Y/N and type [ranch or Italian]) will be recorded and standard serving sizes (1oz) applied. Schools use a 24oz bottle of each dressing and monitor usage in daily production records. Per the school district, 25–35% of students use salad dressing (based on the assumption that 1oz is used per child), which is consistent with objective assessments in prior research [29]. Further, the district’s salad bar coordinators reported that over-portioning has not been an issue, likely due in part to close monitoring by staff. However, given potential for caloric intake to vary based on salad dressing portion applied, we will monitor usage and adjust rating methods accordingly. Foods brought from home will be noted and will only be rated if the student also purchased a school lunch (i.e., went through the lunch line and had a pre-consumption photograph taken), and if the non-school food is present in both the pre- and post-consumption photograph. At least 20% of images will be double rated and IRRs calculated for each item. If IRRs fall below 0.80, raters will be retrained and images re-rated. We have demonstrated our ability to achieve and maintain extremely high IRRs using these methods [28]. We also validated use of DI against measured weights to estimate volume and waste from salad bars and demonstrated excellent IRRs (ICCs = 0.91) and accuracy for both starting portions (ICC = 0.74) and waste (ICC = 0.98) across vegetables [52].

## Statistical analyses

### Power analysis

Power and effect size of the multilevel models that will be used to analyze the variety of outcomes in our cluster RCT are a function of a number of parameters: (1) the number of clusters (J = 16 independent schools [8 Intervention and 8 Control]), (2) the cluster size (*n* = 295 lunches per school), and (3) the ICC (estimated as 0.01, 0.05, and a much more conservative 0.10), which takes into account the correlated nature of our data and is the ratio of variability between clusters to the total variability [66]. Optimal Design Plus Empirical Evidence v3.01 software [67] was used to calculate the minimum detectable effect size given the parameters above, α = 0.05, and a desired power of 80%. For ICCs of 0.01, 0.05, and 0.10, we have 80% power to detect small to medium effect sizes of 0.17, 0.35, and 0.48, respectively. Thus, our proposed sample of 14,160 observations will be more than adequate to evaluate the study aims.

### Analyses

Prior to analyses, distributions of all measures will be examined. Contingency tables and frequency distributions will be evaluated (for categorical and continuous variables, respectively), and transformations considered. Outliers will be checked for errors before being used in analyses. Sex differences will be assessed for all outcomes; if significant differences are found, analyses outlined below will be stratified by sex. All tests will be 2-sided at α = 0.05 level, corrected for multiple comparisons, and conducted in SAS v9.3.

Analyses are dictated by the cluster RCT study design. Data are hierarchical, gathered in multiple levels. The first level of measurement is from students, clustered within schools, the second level of measurement. We are measuring consumption based on students, yet their treatment assignment is defined by the school’s assignment. This nested structure must be considered in analyses as the assignment of intervention to the schools can result in students within schools being positively correlated for the outcomes. Without accounting for correlated nature of the data, Type 1 error rate for the intervention effect might be inflated and the significance of the study findings misinterpreted. Multilevel Linear Modeling (MLM) is a type of regression used to analyze nested data as it can accommodate fixed and random effects and correlated observations within units of assignment (schools). SAS PROC MIXED (for continuous variables) and PROC GLIMMIX (for dichotomous and polytomous data) have MLM applications which take into account random effects and correlated observations and will be used in these analyses. Potential covariates include grade, NSLP participation, and month of assessment, and factors related to the school cafeteria environment that might differ between schools (e.g., lunch duration), where appropriate. Using these models, we will evaluate differences between Intervention and Controls schools in types and variety of FVs and categories of vegetables selected, and subsequent consumption (% consumed/wasted) at baseline and 4–6 weeks after salad bars are installed. Variety will be recorded as the number of different types of FVs selected for each meal, and the number of categories of vegetables (ranging from 1 to 5; corresponding with USDA categorization) [44]. For the primary outcome (FV consumption), plate waste ratings will be calculated for fruits, vegetables, each specific FV category, and also averaged across FVs. We will also evaluate the effect of location of FVs (salad bar, lunch line, or at the point of sale) and serving type (self-serve, pre-portioned) of each item on FV selection and consumption to inform best implementation practices.

We will evaluate differences in dietary quality (assessed with the HEI) [57] for the total lunch and energy intake (kcals) for the total lunch, FVs, non-FVs, and beverages between Intervention and Control schools. MLM will be applied to examine the association between levels of FV intake and overall calorie consumption at lunch, both independent of school group as well as between Intervention and Control schools, to inform obesity prevention efforts.

Monthly sales (and reimbursements) and NSLP participation data will be characterized using descriptive statistics and graphical approaches. Changes in FV sales (and reimbursements) per student (based on enrollment) and % NSLP participation will be calculated (based on the total number of days lunch was served), evaluated by month. Schools will be compared on these outcomes using paired t-tests (matching on month for each school pair). Responses from cafeteria surveys and process logs will be examined to identify barriers and facilitators and implementation differences related to salad bars to inform sustainability. These variables will also be considered as potential factors explaining any differential findings between schools. Open-ended survey responses will be qualitatively examined for common themes by two independent raters, guided by thematic analysis, to identify, analyze and report themes within and across data [68]. This realistic method allows for meaning to come from participants, rather than from pre-existing codes [69].

To examine the potential differential responses to salad bars among sociodemographic groups, main effects for Title I status and race/ethnicity school group (A through D) will be examined in Aims 1 through 3 analyses. If main effects are observed, models will be stratified by sociodemographic group and moderator analyses considered as appropriate.

## Discussion

This investigation responds to the urgent need to conduct rigorous research on policy and environmental approaches to obesity prevention. It has high public health significance, given the extent of the risks unhealthy dietary intake poses to children, especially those from systematically oppressed and minoritized backgrounds, who are most likely to participate in the NSLP. It also capitalizes on a unique natural experiment by systematically evaluating schools with and without salad bars in a large, diverse district.

The practice of installing salad bars, although intuitively appealing, has advanced well-ahead of the evidence. This investigation will make a significant contribution to policy research by providing the empirical data necessary to evaluate whether salad bars help achieve the HHKFA’s major goal of increasing FV intake within the NSLP [14]. It will also enhance understanding of the role of FVs and salad bars on energy intake at lunch in the NSLP. Moreover, results will identify consequences of and barriers and facilitators to salad bar implementation, including investigating how salad bars impact sales and NSLP participation, informing sustainability efforts. This application improves upon prior research in this area via its rigorous experimental design (RCT); large sample of students nested within matched schools; use of psychometrically sound, objective assessments of dietary intake by blinded assessors; consistent salad bar implementation; and a detailed analysis plan, all of which will minimize the influence of bias on results. Findings will be disseminated via policy briefs and to scientific, school, and community outlets. Only one prior RCT has prospectively examined the effects of salad bars on objectively assessed FV intake, and none has evaluated their effects on energy intake in school lunch. Examination of the potential moderating role of school-level sociodemographic factors further strengthens the significance of this application, enhances its external validity, [70] and will ultimately lead to targeted efforts to enhance dietary intake among children at greatest risk for obesity.

Since the initiation of this study, we have completed collection of Year 1 and the majority of Year 2 data, yielding > 14,000 images of school lunches. Data collection was paused in March 2020 when schools closed due to COVID-19, canceling post-ratings of one pair of schools scheduled to be rated that month. Students returned to school in Fall 2020, yet salad bars have not yet re-opened. The COVID-19 pandemic highlighted the critical role of school meals in addressing food security, and simultaneously placed enormous pressure on school nutrition programs to ensure students were fed [71]. When school buildings were closed in the Spring and Summer of 2020, schools set up meal distribution sites and delivery systems (all while understaffed and under-resourced). The return to school in 2020-21 brought new challenges related to increased demands for school meals, risk mitigation, staffing shortages, and supply chain disruptions. In response, the USDA issued national meal pattern waivers, allowing schools to veer from NSLP nutritional standards—including those related to milk, whole grains and sodium set forth by the HHKFA—and mandated universal free meals [71–73]. Many districts, including the one in this investigation, transitioned to more pre-packaged meals during this time and temporarily eliminated a la carte offerings. Although these temporary waivers helped schools meet the meal demands during that time, there is concern that diet quality has been compromised [71–73]. This is a particular concern given extension of nutrition waivers and the enhanced role of schools in addressing food security [71–73]. There is thus a great need to investigate how these changes have impacted children’s nutrition.

Given that surfaces are not the primary mode of COVID-19 transmission, and because a healthy diet is important to support immune functioning and overall health, some have argued that salad bars should return to schools [74]. However, it is unclear how potential barriers to the re-implementation of salad bars, such as staffing shortages and parents’ fears of the virus, might affect this process. Personal communication with this district’s Food and Nutrition Services stated plans to resume salad bar operations, yet acknowledged facing the same challenges as those encountered nationwide—adjusting to changes in school food policy mandates, supply chain disruptions, and staffing shortages. The resumption of data collection in the current application depends on timing of the return to salad bars in schools. Importantly, however, current estimates suggest that in our two years of data collection, we far exceeded projected numbers, even when removing data from the pair of schools for whom post-ratings were not conducted. To date, we have applied laboratory rating procedures on an estimated 11,000 school lunches across time points, from an estimated 5,500 students. Thus, even if data collection does not resume, we are optimistic that we will be adequately powered to respond to our study aims and hypotheses.

In sum, this study stands to be one of the most definitive investigations of school salad bars to date and will inform school nutrition policies and programming designed to enhance dietary intake and reduce obesity. The vital role of schools in promoting children’s nutrition and preventing food insecurity has become clearer to the nation since the onset of the COVID-19 pandemic, making this work particularly timely [71]. Future directions for this line of research include using these data, combined with results from our formative work, to design targeted interventions (adjunctive or alternative to salad bars) to optimize children’s FV intake within current school policy mandates.

## Data Availability

There are no data in this manuscript. Qualified individuals interested in study materials should contact the primary author (MKB).

## Abbreviations

NSLP: National School Lunch Program
FV: Fruits and Vegetables
